# Addressing obesity in the management of knee and hip osteoarthritis – weighing in from an economic perspective

**DOI:** 10.1186/s12891-016-1087-7

**Published:** 2016-05-26

**Authors:** Anna Flego, Michelle M. Dowsey, Peter F. M. Choong, Marj Moodie

**Affiliations:** Deakin Health Economics, Faculty of Health, Deakin University, 221 Burwood Hwy, Burwood, Melbourne, 3125 Australia; Department of Surgery, University of Melbourne, St. Vincent’s Hospital, Melbourne, VIC Australia

**Keywords:** Obesity, Osteoarthritis, Economic evlaution, Cost effectiveness, Costs, Cost burden

## Abstract

**Background:**

Obesity is one of the only modifiable risk factors for both incidence and progression of Osteoarthritis (OA). So there is increasing interest from a public health perspective in addressing obesity in the management of OA. While evidence of the efficacy of intereventions designed to address obesity in OA populations continues to grow, little is known about their economic credentials.

The aim of this study is to conduct a scoping review of: (i) the published economic evidence assessing the economic impact of obesity in OA populations; (ii) economic evaluations of interventions designed to explicitly address obesity in the prevention and management of OA in order to determine which represent value for money. Besides describing the current state of the literature, the study highlights research gaps and identifies future research priorities.

**Methods:**

In July 2014, a search of the peer reviewed literature, published in English, was undertaken for the period January 1975 – July 2014 using Medline Complete (Ebscohost), Embase, Econlit, Global Health, Health Economics Evaluation Database (HEED), all Cochrane Library databases as well as the grey literature using Google and reference lists of relevant studies. A combination of key search terms was used to identify papers assessing the economic impact of obesity in OA or economic evaluations conducted to assess the efficiency of obesity interventions for the prevention or management of OA.

**Results:**

14 studes were identified; 13 were cost burden studies assessing the impact of obesity as a predictor for higher costs in Total Joint Arthroplasty (TJA) patients and one a cost-effectiveness study of an intervention designed to address obesity in the managment of mild to moderate OA patients.

**Conclusion:**

The majority of the economic studies conducted are cost burden studies. While there is some evidence of the association between severe obesity and excess hospital costs for TJA patients, heterogeneity in studies precludes definitive statements about the strength of the association. With only one economic evaluation to inform policy and practice, there is a need for future research into the cost-effectiveness of obesity interventions designed both for prevention or management of OA along the disease spectrum and over the life course.

## Background

Obesity poses one of the greatest contemporary global public health challenges. Worldwide, the number of overweight or obese individuals has more than doubled since 1980 [1]. With rising prevalence rates in adult populations, the consequential growth in obesity-related chronic disease, including osteoarthritis (OA), is inevitable. Globally, the prevalence of OA, particularly of the large weight-bearing joints such as the knee and hip, is also predicted to grow [2], spurred on as a result of an ageing population but also an ageing population that is getting heavier [2].

There is well established evidence associating obesity (BMI ≥ 30 kg/m^2^ [3]) and OA, with obesity being identified as a major but modifiable risk factor for both OA disease incidence and progression [4–6]. The issue with obesity and OA is that the two conditions often coincide, working synergistically to perpetuate poor function and a greater likelihood of sedentary lifestyles which inevitably lead to higher levels of disability and a reduction in quality of life [7]. Furthermore, the need for total joint arthroplasty (TJA) surgery for the treatment of severe OA in knees and hips is more likely to arise in the obese [8] and earlier in life [9]. Changulani et al. 2007 reported that patients with a BMI of >35 kg/m^2^ who were treated by knee replacement were, on average, 13 years younger than their normal weight counterparts; the authors alluded to the potential implications of younger age for lifetime management of OA [9]. Wang et al. 2013 also highlighted that weight gain and persistent excess weight from early adulthood increased the risk of TJA of the hip and knee for OA [10].

Knee and hip OA is one of the leading causes of global disability and was ranked as the 11th highest contributor to global disability in the most recent Global Burden of Disease study in 2010 [2]. As such, OA as a disease results in large indirect costs to society, mostly driven by impacts on productivity [11]. These indirect costs, when coupled with rapid growth in direct healthcare related costs associated with management of the disease and specifically TJA as a major cost driver [12], mean that the economic burden is huge. Furthermore, it is likely to be exacerbated by the aforementioned growing obesity and OA prevalence. Despite TJA being a very effective and cost-effective treatment option for end stage hip and knee OA, 2013 data from the Organisation for Economic Co-operation and Development (OECD) highlights continuing growth in the number of procedures being carried out with faster growth in knee replacements (TKA) particularly in countries with higher rates of overweight and obesity such as Australia, USA and the UK. It has been estimated that in 2007 in Australia, allocated health care expenditure on OA alone was approximately AUD 2 billion [13]. However, given that obesity rates are likely to continue to rise, this will potentially lead to substantial increases in the prevalence of OA and an even greater burden on health care expenditure [13].

There is now a substantial body of evidence focusing on the relationship between obesity and OA from a variety of perspectives. This includes investigation of the causal relationships of obesity and OA through biomechanical [14], physiological [15] and inflammatory mechanisms [16] and quantification of the impact of obesity on OA outcomes [14]. Attention has also been afforded to the benefits of weight loss in OA populations including a systematic review which concluded that a 10 % reduction in body weight is likely to have positive clinically meaningful effects on OA symptoms such as pain and disability [17]. A recent review focused on addressing obesity in knee OA, identified 9 randomised controlled trials of weight loss interventions in people with knee OA. It concluded that there are several strategies likely to be successful in the management of knee OA [18]. However, the evidence base is far from definitive in terms of what is the most efficacious type of intervention, who it should specifically target and when along the treatment pathway it should be offered. There are the usual challenges of whether obesity interventions can achieve long term weight maintenance and prevent weight regain [19], particularly in a population where OA symptoms may hinder physical activity efforts. There is also some concern that interventions that target weight loss alone may lead to muscle weakness and some bone density loss [20], meaning that an intervention that targets both weight loss and appropriate physical activity simultaneously is likely to be more favourable. This is reflected in current treatment guidelines recommending that weight maintenance and exercise in combination are suitable for managing OA symptoms [21, 22], although, in reality, it is often difficult to determine compliance with these recommendations.

Despite this apparent research momentum, it is not clear what the contribution of research from a health economics perspective has been in terms of supporting the use of obesity interventions in the prevention or management of OA. The discipline of health economics is fundamentally concerned with the allocation of scarce healthcare resources in an environment of competing demands with the goal of maximising a society’s welfare in the process [23]. Health economists typically carry out two different types of studies designed to achieve different purposes. Firstly, they describe and predict the economic impact of a disease or risk factor in order to quantify its cost burden (commonly known as cost of illness studies); secondly, they evaluate the incremental costs and benefits of alternative options to current practice (cost-effectiveness studies) [24] to inform resource allocation decisions. The latter essentially addresses the main objective of economics per se, that being efficiency, or maximising the benefit from available resources.

The goals of this study are two-fold. Firstly, it aims to identify and review the published economic evidence that is focused on describing and quantifying the economic impact of obesity in OA populations. Secondly, it reviews economic evaluations of interventions designed to explicitly address obesity in OA populations in order to prevent the onset of OA or improve OA clinical outcomes and potentially health related quality of life (HrQoL), to determine which interventions represent value for money. In doing so, the study also highlight gaps in the literature and identifies future priorities for this burgeoning area of research.

## Methods

### Literature search strategy

A systematic search of the published peer reviewed literature was conducted in July, 2014 by the lead author. The search was limited to full text literature published in the English language from January 1975 – July 2014. Six electronic databases were searched: Medline complete (Ebscohost), Embase, Econlit, Global Health, Health Economics evaluation database (HEED) and all Cochrane Library databases using the same search terms and Boolean operators in each database. Key words used and search combinations were as follow: osteoarthritis or arthritis or joint replacement or joint prosthesis or arthroplast* or knee arthroplasty or hip arthroplasty AND obes* or obesity or overweight or weight gain or weight loss or BMI or body mass or weight* or weight control AND prevention or treatment or primary prevention or secondary prevention AND economic* or economic evaluation or price or cost or cost-effectiveness analysis or economic benefits or cost benefit analysis or cost burden. A targeted Google search of the grey literature using the search terms was conducted, plus the reference lists of all relevant articles were searched for further studies.

### Study selection

**Fig. 1:**
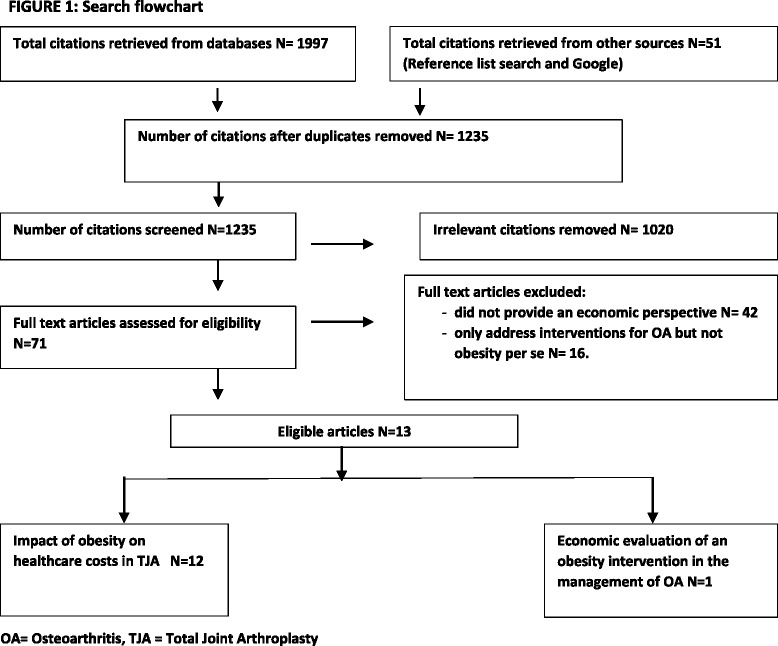
Search flowchart. OA Osteoarthritis, TJA Total Joint Arthroplasty

All relevant abstracts obtained from each database were exported to ENDNOTE, X7 (Thomson Reuters) with duplicate articles removed. Titles and abstracts were searched and relevant full -articles, extracted and reviewed (see flow chart, Fig. 1). For inclusion, studies had to fulfil one (or more) of the three following broad criteria:

1. A costing or cost of illness study which evaluates the association of obesity in any OA study population with healthcare or other societal costs.
2. A partial economic evaluation study which assesses either the costs alone or the costs and outcomes of an obesity intervention in any OA study population but without comparison to an alternative healthcare pathway (cost description or cost-outcome description) or where the costs of alternatives are examined but without consideration of outcomes simultaneously (cost analysis). An obesity intervention was defined as any intervention that included a component that addressed weight loss or weight maintenance in order to impact on OA symptoms and outcomes.
3. A full economic evaluation study such as a cost-effectiveness analysis, cost utility analysis or cost benefit analysis which assesses both the costs and benefits of an obesity intervention against a known alternative or usual care in any OA study population.

The three criteria were purposely broad given that this is a scoping review designed to find any relevant studies and highlight gaps in the literature.

## Results

Figure 1 identifies all steps in the search process and the resulting studies extracted. In the first instance, 2048 citations were retrieved from searched databases (1997) and other sources (51). After removal of duplicates and irrelevant citations, 215 abstracts of candidate articles were screened of which 71 full text articles were assessed for eligibility. The search yielded 12 costing studies (Table 1) and one full economic evaluation (Table 2).

**Table 1:** Studies assessing the impact of obesity on resource use in total hip or knee arthroplasty

Author, Year	Healthcare setting/Country	Study population	Research aim/focus	Measurement of obesity	Costing perspective/measurement and types of counted	Results
Epstein AM, et al, 1987 [25]	Large acute care hospital, USA	278 patients who underwent TKA and 111 patients who underwent THA, October 1983 -September 1984.	To determine the relationship of body weight to LOS and total charges for all patients undergoing THA or TKA	Height and weight taken from pre-operative medical records. 5 levels of weight status categorised by actual weight compared to ideal weight as a %	Health service provider perspective capturing charge data for the inpatient stay only.	Extremely overweight patients (≥ 188 % ideal) had 35 % mean longer LOS (*p* < 0.01) and 30 % higher total charges (*p* < 0.01) than normal weight counterparts. Extremely underweight patients also reported significantly higher costs.
Jibodh SR, et al, 2004 [26]	Large acute care hospital, USA	188 patients who underwent primary THA, 1996 – 2001.	To determine the influence of BMI on perioperative morbidity (time of surgery until discharge) on functional recovery and hospital service use (LOS,total and individual cost items)	Height and weight taken from pre-operative medical records to calculate BMI and categorised into non obese(BMI < 25), mild(BMI >25-29.9), moderate (BMI>30-39.9) and severe (BMI >40)	Health service provider perspective using hospital charge data and reporting total charges and 8 separate billing categories	No significant difference in LOS between 4 BMI groups. A trend towards higher overall charges with increasing obesity but not statistically significant. No significant differences in any of the individual charges were noted between 4 BMI groups in any of 8 billing categories, however morbidly obese patients longer mean operative time (*P* < 0.05)
Vincent HK,et al, 2007 [27]	Inpatient rehabilitation hospital, USA	342 participants who underwent primary or revision TKA, January 2002 - March 2005. Complete case analysis on 285 participants.	To examine the effect of obesity on functional and financial outcomes in patients with TKA undergoing inpatient rehabilitation.	Height and weight taken from patient medical records to determine BMI and categorised as obese (BMI > 30) or non-obese(BMI < 30)	Health service provider perspective using hospital charge data collecting total hospital charges and daily charges for period of inpatient stay only.	LOS was longer in primary and revision obese patients (9.8 days) than for non- obese patients (8.8 days) (*P* < 0.05). Total charges were higher for obese patients (USD 12,386) than non -obese patients (USD 10,618) (*P* < 0.005). Primary TKA group; total hospital charges were significantly higher in the obese than non- obese group (*P* < 0.05)
Vincent HK et al, 2007 [28]	Inpatient rehabilitation hospital, USA	339 obese and non- obese patients with primary or revision THA, January 2002- March 2005. Complete case analysis on 178 participants.	To examine the effect of increasing BMI on functional and financial outcomes in patients with THA undergoing inpatient rehabilitation	Height and weight taken from patient medical records to determine BMI and categorised as non- obese (BMI < 25) overweight (BMI 25-30) obese (BMI > 30-39.9) and severely obese (BMI ≥ 40)	Health service provider perspective collecting total hospital charges and daily charges (using total charges and dividing by LOS) for the period of the inpatient stay only.	LOS were significantly different in the severely obese group compared with the non- obese group (*p* < 0.05). A significant curvilinear relationship between LOS and BMI with the lowest LOS found in overweight and obese persons (*R* squared =0.124 *P* < 0.05). Total charges were greater in the severely obese group compared to the overweight group (P < 0.05).
Vincent HK & Vincent KR, 2008 [29]	15 independent rehabilitation hospitals, USA	5428 obese and non-obese patients who underwent primary TKA or revision TKA, January 2002- March 2006.	To determine the influence of obesity on rehabilitation outcomes including LOS and hospital charges following TKA	Height and weight taken from patient medical records to determine BMI and categorised as non-obese (BMI < 25) overweight (BMI 25-30) obese (BMI > 30-39.9) and severely obese (BMI ≥ 40)	Health service provider perspective with collection of total charges and pharmacy, occupational and physical therapy rehabilitation hospital charges	LOS was longest in the non- obese group compared to all other groups (*P* < 0.05) but age differences amongst groups likely to be impacting on results. The severely obese group had the highest daily charges (USD 36 excess dollars) (*p* < 0.05) but not physical therapy charges or total charges which was highest in the non -obese group (*P* < 0.05). A significant interaction effect was found for TKA status (primary versus revision) and BMI group for total charges (*P* < 0.05).
Batsis JA, et al, 2010 [30]	Large acute care hospital, USA	5539 uncomplicated TKA recipients, 1996- 2004 and classified by BMI (WHO) categories.	To determine the impact of BMI on post-operative outcomes and resource utilization following elective TKA	Height and weight taken at time of surgery and recorded in own joint registry to determine BMI and categorised as BMI normal (BMI 18.5- 24.9) overweight (BMI 25-29.9) obese (BMI > 30-34.9) and morbidly obese (BMI ≥ 35.0)	Health service provider perspective with all direct costs associated with inpatient stay including physician services and readmission within 30 days associated with the primary surgery.	Overall costs were similar among normal, overweight, obese or morbidly obese patients (*P* = 0.24) Post-surgical costs were no different among groups (*P* = 0.44). Higher BMI was associated with a higher mean anaesthesia and operative times and a higher overall Charlson comorbidity index.
Batsis JA, et al, 2009 [31]	large acute care hospital, USA	5642 unilateral uncomplicated THA patients between 1996 -2004 and classified by BMI categories.	To determine the impact of BMI on post-operative outcomes and resource utilization following elective THA	Height and weight taken at time of surgery and recorded in own joint registry to determine BMI and categorised as BMI normal (BMI 18.5- 24.9) overweight (BMI 25-29.9) obese (BMI > 30-34.9) and morbidly obese (BMI ≥ 35.0)	Health service provider perspective with all direct costs associated with inpatient stay including physician services and readmission within 30 days associated with the primary surgery.	No significant differences between BMI groups for LOS, post-operative overall, hospital and physician costs. Operative and anaesthesia costs were higher in morbidly obese group than all other groups. All other adjusted costs were non-significant. No significant differences between groups in: composite 30 day endpoints, rate of patient transfers to ICU or number of days in ICU.
Kim, SH, 2010 [32]	Short stay, community hospitals in the Nationwide Inpatient Sample (NIS- 2006), USA	229 001 primary TKA recipients and 497 001 primary THA recipients in the USA captured in the NIS.	To estimate the prevalence of morbid obesity (≥40 kg/m2 in the THA and TKA sample and to determine if there is greater resource use attributable to morbid obesity for primary TJA	Presence of obesity (BMI ≥30.0) and morbid obesity (BMI ≥ 40.0) identified by the corresponding ICD_9M codes for obesity in hospital administrative databases	Health service provider perspective using hospital inpatient charge data converted to cost data and reporting on overall hospital costs only	When adjusted for known confounders, hospital resource consumption for primary THA and TKA was 9 % and 7 % higher among morbidly obese than among non-obese patients respectively
Dowsey M, et al, 2011 [33]	Large acute hospital, Australia	521 primary TKA recipients, January 2006 - December 2007.	To determine whether obesity was independently associated with higher hospital costs for the index procedure and over the following 12 months.	Presence of obesity (BMI ≥30.0) captured from preoperative measures recorded in own hospital joint registry	Healthcare service provider perspective capturing total inpatient costs for the index TKA, relevant readmissions in the first 12 months and the two together named episode of care.	Statistically significant association between obesity and higher inpatient costs ($1127 *P* = 0.036) and higher episode of care costs (+1,821 *P* = 0.024). Using BMI as a continuous variable, cost of index procedure increased by $129 and episode of care costs increased by $159 per unit increase of BMI.
Silber JH, et al, 2012 [34]	47 acute hospitals of varying size across multiple locations, USA	2045 obese patients (BMI ≥ 35 kg/m2) matched to non-obese patients undergoing THA, TKA (primary or revision), colectomy, thoracotomy, 2002- 2006. 75 % of the sample underwent TJA.	To study the medical and financial outcomes associated with surgery in the elderly obese.	Presence of severe obesity (BMI ≥ 35.0 < 40.0) and morbid obesity (BMI ≥ 40.0) captured from baseline BMI data in hospital medical records	Healthcare service provider perspective using 2 alternate costing methods (Medicare payments versus costs using cost to charges ratios) (to determine overall hospital costs from admission to 30 days post operation.	Medicare payments were 3 % greater (*P* < 0.001) and provider costs were 10 % greater for obese compared to non- obese matched counterparts (*P* < 0.001). The Obese group recorded a 12 % longer LOS than their complete matched non obese counterparts (*P* < 0.001)
Maradit Kremers H, et al, 2014 [35]	Large acute care hospital, USA	8129 patients who underwent 6475 primary TKA and 1654 revision TKA, January 2000 - September 2008.	To examine the relationship between obesity, length of stay and direct medical costs during the index hospitalisation and a 90 day window taking into account obesity related co-morbidities.	Height and weight taken from patient admission records to calculate BMI and categorised into 8 BMI categories and as a continuous variable	Health service provider perspective using hospital administration databases and converting charges to costs using cost centre specific ratios. End points of hospital LOS, direct medical costs during hospitalisation and total medical costs during the 90 day window	LOS was longer at the extreme ends of the BMI spectrum only with mean LOS lowest in those with BMI 30-40.0. After adjusting for known confounders, every 5 unit increase in BMI over 30 was associated with higher mean costs of USD 421 for hospitalisation and USD 524 for 90 days and remained significant after adjustment for comorbidities (*P* = <0.001) and complications (*P* = 0.004).
Maradit Kremers H, et al, 2013 [36]	Large acute care hospital, USA	8973 patients; 6410 primary THA and 2563 revision THA's, January 2000 - Sept 2008.	To examine the relationship between obesity, length of stay and direct medical costs during the index hospitalisation and a 90 day window taking into account obesity related co-morbidities.	Height and weight taken from patient admission records to calculate BMI and categorised into 8 BMI categories and as a continuous variable	Health service provider perspective using hospital administration databases and converting charges to costs using cost centre specific ratios. End points of hospital LOS, direct medical costs during hospitalisation and total medical costs during the 90 day window	Increasing BMI was associated with higher hospital costs and this association persisted among patients without significant comorbidities or complications. After adjusting for known confounders, every 5 unit increase in BMI was associated with USD 744 and USD 1183 higher hospitalisation and 90 day costs respectively. (This corresponds to about 5 % higher hospitalisation and 90 day costs respectively).

*TKA* total knee arthroplasty, *THA* total hip arthroplasty, *TJA+* total joint arthroplasty, *LOS* length of stay, *BMI* body mass index, *USD* USA dollars, *NIS* national inpatient survey, *ICD-9 M* The International Classification of Diseases, 9th Revision

**Table 2:** Economic evaluation of obesity interventions in OA populations

Author, Year, Country	Intervention	Target population	Type of economic evaluation, time horizon	Costing perspective, costs included, base year for costing	Outcome measurement	Costs	Cost- efficacy
Sevick MA, et al, 2009 [38]	18 month dietary and exercise intervention in overweight/obese elderly patients with knee OA The ADAPT trial - 4 arms in the trial: healthy lifestyle control, diet, exercise, exercise and diet.	participants aged ≥ 60 year, BMI ≥ 28 kg/M^2,^with radiographic evidence of knee OA (but not advanced stage radiographic evidence)	Cost-efficacy study over 18 months; no modelled analysis.	Managed care organisation payer perspective. Intervention costs (staff time, facilities, equipment and materials) collected prospectively and self- reported health services consumed by participants over the duration of the trial. All costs adjusted to Yr. 2000 USD	WOMAC (function, pain, stiffness components separately), weight change, 6 MWT and stair climb.	Total intervention costs and health service utilisation costs in USD per participant per month: control: $32, Diet only: $160, Exercise only: $152, Exercise and Diet:$304	Exercise and diet intervention most cost effective for improved self-reported function, pain and stiffness (USD 24 per PPI in function, USD 20 per PPI in pain, USD 56 per PPI in stiffness) compared to healthy control. Diet arm was most cost effective for reducing weight (USD 35 per PPR in baseline body weight)

*ADAPT* arthritis, diet and physical activity promotion diet, *BMI* body mass index, *WOMAC* Western Ontario and McMaster Universities Arthritis Index, *6 MWT* 6 min walk test, *USD* US dollars, *PPI* percentage point improvement, *PPR* percentage point reduction, *OA* osteoarthritis, *PPI* percentage point increase

### Impact of obesity on resource use studies in TJA populations

#### Settings and target group

There were twelve studies identified as cost burden studies; they all included analysis of the association between the presence of obesity in total joint replacement populations used as a predictor of healthcare costs [25–36]. Nine studies were set in acute care hospitals with a focus on THA [26, 31, 36] or TKA recipient populations [30, 33, 35] or included both THA and TKA recipients [25, 32, 34]. The remaining three studies were set in inpatient rehabilitation hospitals with two studies focusing on TKA recipients [27, 29] and one on THA recipients [28]. Table 1 summarises the main characteristics of the identified studies. They included a mix of studies focusing on primary or index TJA surgery only [25, 26, 30–33] or inclusion of both primary and revision types of surgeries [27–29, 34–36]. This highlights the heterogeneity between studies in relation to the specific populations under study, with each having its own unique set of patient inclusion and exclusion criteria. Nearly all studies were conducted in the USA with only one study conducted in Australia [33].

#### Costs

All studies, regardless of setting, were conducted retrospectively from a narrow healthcare perspective and were restricted to the collection of direct medical costs or charges specific only to that setting. All studies relied on hospital administration data. While this enabled most of the studies to have near complete data, the downside was that the costs reported in each study were limited and did not reflect the total cost burden. As an example, the studies conducted in the acute care setting took no account of costs of the resources consumed in rehabilitation, supportive nursing facilities, outpatient and ambulatory care services or costs borne either by other sectors or by patients themselves. Methods used for identification, measurement and reporting of cost outcomes were heterogeneous and not always transparent; some studies provided little detail about their costing methodology including the selection or specification of cost items included in reported aggregate resource use [27, 28, 32] or the reference year used for the costing [27, 28]. Some studies only presented hospital charges rather than costs per se [26–29], the former usually reflecting an element of profit as well as a method of recouping uncompensated costs. If this profit is considered above and beyond the societal opportunity cost, then adjustments should be made with cost to charge ratios [37]. However the choice of ratio applied (eg. the use of an overall hospital ratio versus a department specific ratio) varied between studies and introduced an element of uncertainty in the estimates obtained. The timeframe over which resource use was measured also varied greatly between studies. Some studies only accounted for hospital resource use during inpatient stay post -surgery [25–29, 32]. Others sought to capture resource use related to potential readmissions by measuring up to 30 days [30, 31, 34] or three months post -surgery [35, 36]; only one study followed patients for 12 months post- surgery in order to capture a picture of the annual hospital resource use as impacted by the presence of obesity [33].

#### Obesity measurement

The studies varied in terms of ascertaining and classifying the presence of obesity in the study population. In Epstein et al [25], the earliest study, obesity was measured as the relative weight to normal weight which is now considered an outdated method for classifying obesity; all the other studies used Body Mass Index (BMI) categories and/or BMI as a continuous variable although there was variation between studies as to the number and cut offs for the BMI categories used. Nearly all identified studies used recorded height and weight data obtained from medical records as the basis of BMI calculations. However the Kim et al. study that analysed a sample of 229 001 TKA recipients and 497 001 THA recipients captured in the US Nationwide Inpatient sample (NIS), used hospital administration coded data to determine the presence or absence of obesity rather than the height and weight recorded body measurements of patients per se [32]. This opened the study to misclassification bias and it has also been suggested in the literature that administrative data underreports the presence of obesity [36].

#### Analytical approach

The main variation in analytical approach between studies was whether an analysis conducted accounted for the presence of obesity related co-morbidities as a potential mediator or confounder between obesity and hospital costs. More recent studies were more likely to present results of both unadjusted and adjusted analyses using well known comorbidity indexes to show both the mediated and independent effect of obesity on hospital costs. Kremers et al [35], stated that obesity is a risk factor for several costly comorbidities and, therefore, controlling for co-morbidities may result in underestimation of the true incremental cost of obesity because costs attributable to comorbidities theoretically can be considered attributable to obesity.

#### Results

Given such heterogeneity in elements of study design, including study setting, sample size, population under study, measurement of key variables and the analysis performed, it is not surprising that results of those studies are somewhat mixed. Three studies suggest there is no difference between groups by BMI categories on hospital costs in the acute care setting [26, 30, 31]. The remaining six acute care setting studies found a statistically significant association between the presence of severe obesity (>35 kg/m^2^) and higher health care costs/charges with the difference mostly in the range of 5-10 %. Overall, the more recent studies, with larger sample sizes and longer follow up periods tended to present positive association results. The main driver of the cost difference was overall hospitalisation costs, although length of stay (LOS) related to the primary episode of care, was not always identified as a contributing factor to these higher costs.

Whilst Epstein et al. reported 30 % higher total charges and 35 % mean longer LOS for persons classified as extremely overweight (body weight ≥ 188% of ideal) compared to their normal weight counterparts [25], the costs presented were high and less relevant today given the recent introduction of more streamlined and efficient clinical care pathways.

The two single centre rehabilitation studies [27, 28] reported that severe obesity in both THA and TKA populations was associated with higher overall hospital charges. However, results of the much larger multicentre trial in the TKA population contradicted the results of the single site study by reporting that whilst the severely obese had the highest daily charges of all BMI groups (albeit modest at USD10-36 per day), total charges were highest in the non obese group. The authors cautioned interpretation of these results, given that the average non obese patient was 11.2 years older than those in the severely obese group, indicating that age-related changes could be driving the costs. The authors also reported a significant interaction effect between high BMI and revision surgery leading to prolonged and more costly inpatient care.

### Cost-effectiveness studies

Results of the literature search revealed only one full economic evaluation. The latter was a within trial cost-effectiveness analysis (CEA) study by Sevick et al, 2009 [38], run alongside the ADAPT trial (Arthritis, Diet and Physical Activity Promotion Trial) [39] which followed 316 participants with moderate knee OA and baseline BMI of ≥ 28 kg/m^2^, randomised to one of 4 arms: healthy lifestyle control, diet alone, exercise alone or diet and exercise interventions delivered over 18 months. Key study design characteristics and results of this economic evaluation are summarised in Table 2.

The study provides evidence that a multimodal intervention incorporating both physical activity and diet components is the most efficient choice for improving self-reported function, pain and stiffness as measured using the 3 Western Ontario and McMaster Universities Osteoarthritis Index (WOMAC).

Sub-scales at 18 months compared to a healthy lifestyle control or diet and exercise interventions alone. However, it was not the most efficient choice for weight loss alone or for functional measures of mobility alone. The authors suggest that the magnitude of change in WOMAC scores found in the exercise and diet group could be considered clinically significant in similar Knee OA populations [38]. Despite the reported comparative efficiency of the multimodal intervention arm, the total costs of the ADAPT intervention arms were higher than other CEAs of exercise and other conservative interventions in general OA populations [40–43]; this reflects the resource intensive nature of the intervention design and delivery over the 18 month trial duration. While the authors concluded that both weight loss and physical activity are needed to achieve improvements in subjective physical function and pain, it is not clear whether a diluted approach would necessarily achieve the same results.

There are some limitations to this study that are acknowledged by the authors. Costing was performed from a narrow managed care as health care provider perspective. For example, it did not include patient out of pocket costs such as the cost of pharmaceuticals which are likely to be impacted, particularly if pain and function is improved by the intervention. Other costs that would be included from a societal perspective such as productivity impacts were likewise excluded. The time horizon was also limited to the duration of the trial. Longer term follow up of both costs and outcomes would have provided greater understanding of whether the intervention could maintain function in the long term, prevent disease progression and the associated impact on healthcare resource use over this time. The study population for this evaluation was overweight and obese patients with knee OA but not end stage OA with an average baseline BMI of ≥ 28 kg/m^2^. However exercise maybe more difficult in end stage OA populations and there is also debate about whether exercise is actually beneficial at higher BMI’s (≥35 kg/m^2^) until weight loss is first achieved [4].

## Discussion

Despite a growing body of literature highlighting the importance of addressing obesity in OA populations, this review highlights the current paucity of economic evaluation research conducted in this area. Therefore, it remains unclear as to whether it is efficient to address obesity explicitly for the prevention or management of OA, how to best do it, which population to target, and when along the disease spectrum this should occur.

Nearly all studies identified were cost burden studies in TJA populations conducted to assess whether obesity leads to higher healthcare sector costs. It should also be noted that all of identified the studies related to OA in knees and hips, despite the search of OA being much broader. All of the costing studies adopted the narrow perspective of the individual healthcare setting from which the main cost data were derived; therefore, they did not account for costs associated with the whole continuum of care for TJA and only provided a snapshot of the potential economic impact compared to if a societal approach had been taken. With greater streamlining of acute health care clinical pathways for joint replacement recipients, some of the traditional post-operative costs have shifted away from the acute care setting and onto inpatient rehabilitation, outpatient, community and ambulatory care settings as well as patients and families themselves. Ideally, resource use should be tracked throughout the whole episode of care, using database linkage to gain a broader understanding of the costs associated with the presence of obesity in TJA populations. A Canadian study by Tarride et al [44] which tracked total healthcare resource use in both hospital and ambulatory care settings (though not OA attributable costs per se), demonstrated that healthcare costs per year to OA patients were nearly double those of non OA control participants. A subsequent finding that was not the primary objective of the study was that costs were more than triple for obese OA patients compared to non-obese controls; this highlights that the co-existence of obesity and OA possibly augments the excess financial burden of OA.

Not all of the costing studies reviewed concluded that higher costs in TJA populations were due to obesity per se. However, the more recent studies with stronger methodologies trended towards showing significant differences in healthcare costs between the severely obese compared to other BMI categories, which suggests that there are potential health care cost savings to be realised if weight loss in the severely obese is addressed prior to TJA surgery. Yet focusing only on the cost side of the equation simply identifies a potential problem, but does not address issues of technical (“how to do”) or allocative efficiency (“what to do”) in terms of choosing alternate healthcare programme options; these can only be addressed by economic evaluation studies. Similarly, establishing and subsequently investing in interventions with proven efficacy alone without consideration of the cost implications will inevitably drive healthcare spending upwards [45]. For example, there is increasing interest in the use of laparoscopic adjustable gastric banding surgery, for severely obese (BMI ≥ 35 kg/m ^2)^ to achieve rapid weight loss prior to TKA [46] given some evidence of negative health outcomes post TKA for these patients such as higher rates of infection [47], higher rates of revision [47], and worse pain and function post operatively [48]. Lap band surgery is highly efficacious [49, 50] and cost-effective [50, 51] as a weight loss intervention in the severely obese but it is also a very costly intervention. It remains to be seen whether the incremental cost of adding this procedure prior to TJA in this population is outweighed by the incremental benefits achieved through cost offsets and improved health outcomes including health related quality of life gains immediately post-surgery and over longer time periods. It is certainly worthy of further investigation however, given evidence of the association of pre-operative obesity predicting additional weight gain post TJA [52] which is only likely to further precipitate the ill effects of OA and obesity in combination.

Only one economic evaluation was identified in this review [38]. This showed that despite a small but growing number of efficacy trials that have been conducted to assess the impact of interventions to address obesity in OA populations [18], published cost-effectiveness studies are still lacking as in many areas of obesity research. The Sevick et al study, provided some evidence that weight loss and exercise in combination was most cost-effective in improving pain and function in overweight and obese patients with moderate knee OA compared to only exercise or weight loss independently. Yet the study provides no indication of the intervention’s longer term cost-effectiveness beyond the 18 month trial duration. It would be of particular interest to a range of stakeholders (clinicians, funders and patients) to know if the intervention was able to slow disease progression, control symptoms and maintain function for longer in persons with obesity and OA and subsequently delay their demand for more intensive and costly healthcare that coincides with higher levels of disability. Longer term cost-effectiveness studies however are reliant on either longer duration efficacy studies being conducted or, alternately, extrapolation of outcomes through modelling; the latter can be very informative but are dependent on assumptions especially in relation to maintenance of program effect. It would also be interesting to see how changes in OA symptoms as a result of an intervention manifests in terms of the quality of life impact using a utility-based HRQoL measure that can be used in economic evaluation to produce a cost per Quality Adjusted Life Year (QALY). QALYs provide a common generic measure of benefit taking into account both mortality and morbidity impacts and importantly, within this context, may capture benefit from weight loss and exercise that extends beyond that of the effect on immediate OA related outcomes. This metric also facilitates comparison of the cost effectiveness of interventions within and across disease areas. Even if studies have not incorporated a generic utility based HRQoL measure, there are reliable mapping techniques available. For example mapping techniques have been used to map disease specific measures such as the WOMAC to HRQoL scores on measures such as the EQ-5D [53] and the Assessment of Quality of Life (AQoL) [54] for Australian populations, although there are some limitations to the applicability of such mapping exercises [54].

With only one published cost-effectiveness study to inform policy and practice in this area, it is evident that there is a need for more economic evaluations of interventions to be conducted. This is inherently reliant on a growing evidence base of the efficacy of such interventions which ideally would incorporate economic evaluation alongside such efforts. Modeling could then be utilised to predict longer term cost-effectiveness over the life course and OA disease progression; this would require explicit statements of the assumptions about maintenance of program effect and their testing in sensitivity analysis. Economic evaluation in addressing obesity in OA should ideally adopt a societal perspective given that both costs and benefits may be incurred beyond the heath-care sector and where possible, an incremental cost per QALY should be reported to provide comparison across disease areas.

The notion of intervening to alter disease progression, delay, avoid or reduce complications post TJA by addressing obesity explicitly is, prima facie, likely to have both downstream economic and health benefits to healthcare systems, individuals and society as a whole. In practice, guidelines identify the need to focus on obesity in the management of OA, however it is evident that there is a lack of data both from an efficacy and cost-effectiveness perspective to support and to guide to whom, how and when this should be conducted and whether it should be explicitly incorporated into clinical pathways and public health practice. More research is therefore warranted in order to guide clinicians, funders, decision makers and patients alike.

## Conclusion

Overall, this review has demonstrated that while the presence of obesity in OA populations, and in particular severe obesity, has been shown to have significant health and economic impacts, there is a dearth of evidence from an economic perspective to guide resource decisions about whether addressing obesity explicitly is considered value for money.

## Abbreviations

AQoL, assessment of quality of life; AUD, Australian dollars; CEA, cost-effectiveness analysis; HRQoL, health related quality of life; ICD-9 M, international classification of diseases – 9^th^ revision; LOS, length of stay; NIS, Nnationwide inpatient sample; OA, osteoarthritis; OECD, organisation for economic cooperation and development; THA, total hip arthroplasty; TJA, total joint arthroplasty; TKA, total knee arthroplasty; USD, United States dollars; WOMAC, Western Ontario and McMaster Universities Osteoarthritis Index
